# A Coupled Human and Natural Systems Framework to Characterize Emerging Infectious Diseases—The Case of Fibropapillomatosis in Marine Turtles

**DOI:** 10.3390/ani13091441

**Published:** 2023-04-23

**Authors:** Costanza Manes, Raymond R. Carthy, Vanessa Hull

**Affiliations:** 1 Department of Wildlife Ecology and Conservation, University of Florida, Gainesville, FL 32611, USA; 2 One Health Center of Excellence, University of Florida, Gainesville, FL 32611, USA; 3 U.S. Geological Survey, Florida Cooperative Fish and Wildlife Research Unit, University of Florida, Gainesville, FL 32611, USA

**Keywords:** wildlife, infectious diseases, anthropogenic effects, conservation, EIDs, CHANS

## Abstract

**Simple Summary:**

In a fast-changing world, it is highly important to consider the possible consequences of human-driven effects that can alter natural ecosystems. In this review, we built a framework focused on risk factors associated with new wildlife diseases, such as immune system suppression, pathogen transmission between different animal species (also called “spillover”), and disease spread. Our conceptual framework describes major potential interactions between humans and nature that might occur in coupled human and natural systems, those systems where humans and wildlife are tightly linked, conceptually and geographically. Such interactions consist of certain anthropogenic effects (such as pollution, climate change, unsustainable farming, and more) which we distributed across our conceptual framework to identify their relevance to the wildlife infectious disease risk factors that this manuscript examines. Our developed framework can be applied to many new wildlife diseases, and we provide an application example with an emerging tumoral disease of marine turtles, called Fibropapillomatosis. Our work shows how crucial it is to analyze conservation issues beyond what is immediately apparent, and for science to operate through research collaboration and synergy.

**Abstract:**

Emerging infectious diseases of wildlife have markedly increased in the last few decades. Unsustainable, continuous, and rapid alterations within and between coupled human and natural systems have significantly disrupted wildlife disease dynamics. Direct and indirect anthropogenic effects, such as climate change, pollution, encroachment, urbanization, travel, and trade, can promote outbreaks of infectious diseases in wildlife. We constructed a coupled human and natural systems framework identifying three main wildlife disease risk factors behind these anthropogenic effects: (i) immune suppression, (ii) viral spillover, and (iii) disease propagation. Through complex and convoluted dynamics, each of the anthropogenic effects and activities listed in our framework can lead, to some extent, to one or more of the identified risk factors accelerating disease outbreaks in wildlife. In this review, we present a novel framework to study anthropogenic effects within coupled human and natural systems that facilitate the emergence of infectious disease involving wildlife. We demonstrate the utility of the framework by applying it to Fibropapillomatosis disease of marine turtles. We aim to articulate the intricate and complex nature of anthropogenically exacerbated wildlife infectious diseases as multifactorial. This paper supports the adoption of a One Health approach and invites the integration of multiple disciplines for the achievement of effective and long-lasting conservation and the mitigation of wildlife emerging diseases.

## 1. Emerging Wildlife Diseases in Coupled Human and Natural Systems

Globally, the number of emerging infectious diseases among wildlife has undergone an unsustainable increase in recent decades [1]. From 2000 to 2014, up to 70 new wildlife diseases have been identified across various animal taxa, such as chytridiomycosis in amphibians, AH5N1 avian influenza in birds, and devil facial tumor disease in Tasmanian devils [2]. The increase in wildlife diseases can be attributed in part to an increase in frequency and magnitude of anthropogenic activities and effects at local, national, and global scales [3]. In our work, we identify a new category of anthropogenically exacerbated wildlife diseases, where mechanisms explaining disease spread go beyond the basic principles of virology and epidemiology, as a variety of unseen factors dictate disease outbreak and severity. This category encompasses some newly emerging diseases of wildlife, where dissemination and outbreaks have occurred simultaneously in response to drastic human-driven environmental changes. The challenge of living in an increasingly interconnected human/animal world is that many emerging issues often do not have a clear root. Interactions and dynamics between the different components are increasingly complex, and it has become more difficult for researchers to identify a single cause for a given conservation challenge.

**Figure 1:**
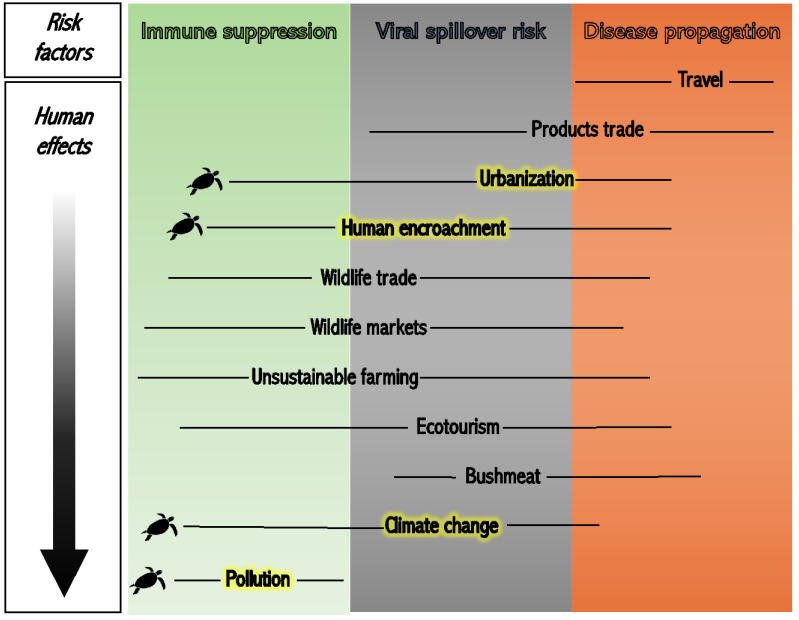
A Coupled Human and Natural Systems conceptual framework to characterize wildlife Emerging Infectious Diseases. Our framework identifies three major risk factors associated with emerging infectious wildlife disease outbreaks: immune suppression (green box), viral spillover risk (grey box), and disease propagation (orange box) (top–left to right). Selected direct and indirect anthropogenic activities (from Travel to Bushmeat) and the effects of those activities (Climate change, Pollution) are listed top to bottom along the framework as indicated by the arrow on the right. The black lines applied to each anthropogenic activity and effect reflect the extent to which each risk factor applies to each of the listed human effects. Some human activities and effects are associated with multiple risk factors and expand horizontally across multiple color blocks. The yellow highlight and black turtle figure are used to indicate specific anthropogenic activities and effects identified as applicable to our case study of sea turtle Fibropapillomatosis.

Coupled Human and Natural Systems (CHANS) is a commonly used concept in interdisciplinary ecology to describe human/animal systems. CHANS are intricate systems involving the participation and interactions between the human and natural dimension [4,5]. Emerging Infectious Diseases (EIDs) are an important part of CHANS, though not fully recognized until recently [6]. CHANS comprise tight linkages between the anthropogenic and the natural world. Understanding these interactions is important for informing conservation because disruption and alteration of certain dynamics within CHANS can create a cascade of effects leading to negative outcomes, such as outbreaks of infectious diseases in wildlife. In our novel framework (Figure 1), we identified three principal wildlife EIDs risk factors: (i) immune suppression, (ii) viral spillover, defined in the EID literature as the jump from an infected species to a new species [7], and (iii) disease propagation. These factors all result at least to some extent from selected anthropogenic activities and effects present in CHANS, namely travel, products trade, urbanization, human encroachment, wildlife trade, wildlife markets, unsustainable farming, ecotourism, bushmeat, climate change, and pollution. This review will explain the epidemiological dynamics by which our framework’s detected risk factors apply to these anthropogenic activities and effects and how each of those can potentially drive outbreaks of EIDs. We then demonstrate its utility by applying our CHANS framework to the case of Fibropapillomatosis (FP) in marine turtles.

### 1.1. Increased Movement: Travel and Products Trade

Travel is linked in our framework to increased risk of disease propagation (Figure 1). There is a clear association between travel and infectious disease spread [8]. The current pace at which humans travel has the potential to transform local outbreaks into pandemics [9]. Moreover, travel is gradually becoming more affordable with the entry of low-cost flights, allowing a greater portion of the world’s population to fly [10]. In this context, it is important to note that humans do not travel alone but carry along the pathogens that they harbor. Alongside human travel, trade of products has also become easier, associated in our framework with increased risk of viral spillover and disease spread (Figure 1). In this era of globalization, products are in continuous movement across continents [11]. Products are considered possible ‘vehicles’ of introductions, since accidental animal transfer with products can lead to invasive species movements, as in the case of spider species traveling alongside fruit, plants, and packaging materials [12,13]. Animals may also transport their respective pathogens when they travel, subsequently introduced in naïve populations [14]. A naïve population in epidemiology indicates one that has not yet been exposed to a certain pathogen and could likely experience infection, since its immune system has not yet adapted to it, increasing the likelihood of a spillover event. Thus, product trade can also contribute to wildlife disease introductions [15].

### 1.2. Anthropogenic Presence: Urbanization and Human Encroachment

Urbanization and human encroachment are linked with both spillover and disease propagation risk factors from our CHANS framework (Figure 1). Human population growth increases the need for space, and areas that were once pristine become semi-urban, and semi-urban areas in turn become urban [16]. Urbanization is thus a type of disturbance that alters ecosystem dynamics [17]. In the last few decades, a greater number of people have been moving into urban areas [18]. As of 2016, 54% of the global population (about 4 billion people) lived in urban areas [19]. Urbanization brings along large-scale human activities and more crowded and dense living conditions, which increase the risk of disease transmission [20,21]. The dense living conditions of urban centers also place multiple hosts and pathogens in close proximity, creating fertile ground for viral spillover events [22]. Similarly, human encroachment is another relevant factor when investigating EID outbreaks [3], potentially altering disease propagation and frequency of spillover effects. This process often results in the reduction of wildlife habitat, forcing wildlife to now seek food and shelter in closer proximity to human establishments. This shift brings people and wildlife in close contact and pushes new species and/or naïve hosts closer together, increasing the flow of pathogens and the chance of spillover effects [23]. Examples of human encroachment effects on wildlife are deforestation and land conversion that result in habitat loss to make space for anthropogenic settlements [23,24]. For example, there is evidence that outbreaks of the Nipah epidemic in Southeast Asia originated from viral spillover from the flying fox (*Pteropus* spp.) to humans. This spillover event was partially attributed to the loss of the bats’ natural habitat following human encroachment in the area [25]. Moreover, the immune suppression risk factor identified in our framework also applies to both urbanization and human encroachment (Figure 1). An increased and prolonged stress exposure can suppress the immune system and make individuals more susceptible to contracting diseases [26]. Urban wildlife is reported to be under higher physiological stress compared to non-urban wildlife, affecting their health and disease susceptibility [27]. The continued encroachment of humans can similarly stress wildlife on multiple levels. Unsuitability or loss of habitats can contribute to physiological stress and compromise immune function [28]. Diet alteration as humans encroach on natural habitats can furthermore lead to health issues in wildlife living in proximity to human settlements in situations of urbanization and/or encroachment [29].

### 1.3. Animal Use: Wildlife Trade and Markets, Unsustainable Farming, Ecotourism, Bushmeat

The three risk factors identified in our framework all apply to the human practices of wildlife trade, wildlife markets, unsustainable farming, and ecotourism (Figure 1). Wildlife trade has previously been indicated as a main driver behind the outbreak of numerous EIDs [30,31]. Wildlife trade as considered here refers to animals taken from their natural habitat and used for sale and trade. During trade and smuggling, live animals are in high stress conditions, kept in close proximity, and experience an increased chance of contact with humans and other species [32]. Wildlife trade is partially responsible for the outbreak of Chytridiomycosis in amphibians, which led to the extinction of hundreds of frog species [33]. In the sale portion, wildlife trade often occurs in wildlife markets, which mix different species, dead and alive, in a relatively small space. This situation allows the flow of multiple and novel pathogens among humans and animals packed in one space and can often be a source of zoonotic infectious diseases outbreaks [34,35]. Thus, both wildlife trade and market scenarios entail risk factors of immune suppression (high stress conditions), disease propagation (proximity), and viral spillover (contact with humans and other species), as shown in our framework (Figure 1). Crowded conditions of unsustainable farming practices present a similar disease propagation and viral spillover risk scenario [36]. Unsustainably farmed animals are typically kept in close contact and experience elevated stress levels due to difficult living conditions. At the same time, the proximity to human handlers and contact with bodily fluids increases disease transmission risk [37]. Farmed animals can therefore harbor pathogens, because of immune suppression and close contacts, and represent a risk to wildlife as well. Peste des Pestis Ruminants outbreaks, for example, have devastated populations of endangered wild ungulates as an effect of spillover from farmed sheep and goats [38]. Unregulated wildlife ecotourism also enables the proximity of humans and wildlife, which can cause physiological stress on wildlife (immune suppression risk factor) and facilitate the spread of diseases (disease propagation risk factor). In this context, it is important to consider that humans can transmit novel diseases to other species as well (spillover risk factor), especially to non-human primates, given the genetic proximity [39]. Bushmeat also plays a role in the emergence of novel diseases [40,41]. We have identified its association to the risk factors of viral spillover and disease propagation in our framework (Figure 1). Bushmeat is defined as the meat of wildlife that is consumed by people, sometimes considered a delicacy, and other times a necessity in the case of protein need in the diet of low-income households, particularly in West and Central Africa [42]. However, the practice of hunting, preparing and consuming bushmeat exposes people and wildlife to risks of viral spillover and disease spread. For example, the first Ebola outbreak started in 1996, when Booué hunters initially contracted the novel virus from the wild [43]. Later re-emergence of Ebola in Congo in 2007 was also connected to the purchase of bat carcasses freshly killed by local bushmeat hunters [44]. Similarly, although we have found no reports to our knowledge, bodily fluids of wildlife carcasses in the environment as a result of bushmeat hunting may still pose a risk scenario of disease exposure and viral spillover to local wildlife species.

### 1.4. Environmental Alterations: Climate Change and Pollution

We connected climate change with disease propagation, viral spillover risk and immune suppression in our CHANS framework (Figure 1). Disease transmission depends heavily on environmental factors, such as temperature, precipitation, sea level elevation, wind, and daylight duration. Thus, disturbances occurring on a broader ecosystem scale can also drive the spread of new wildlife infectious diseases [45]. Climate change is causing rapid and extreme environmental variation and has a major role in disease emergence in wildlife [46]. In 2015, for example, uncommonly high levels of temperature and humidity in Kazakhstan were responsible for the *Pasteurella multocida* type B driven die-off of over 200,000 Saiga antelopes (*Saiga tatarica tatarica*), leading to >60% loss of their global population [47]. Increased climatic suitability for disease vectors or naïve species can also cause increased risk of disease transmission and spillover. In the case of Ross River virus disease in Southeast Australia, extremely heavy rainfalls likely facilitated the mosquito-mediated spread considered responsible for unusually large outbreaks of the disease, which is found to infect various marsupial and ungulate species [48,49]. Fluctuations outside of normal temperature ranges and consequent thermal stress can cause physiological stress and immune alteration in wildlife, with negative impacts on their health [50]. We linked pollution with the immune suppression risk factor in our CHANS framework (Figure 1). Environmental pollution can also contribute to disease outbreaks, in both animals and humans, as exposure to contaminants can alter immune response and increase vulnerability to pathogens [51,52,53,54]. Pollutants can have a direct toxic effect on wildlife immune function, via killing of host tissues and cells, as well as compromising development and functioning of antibodies, leukocytes, and cytokines [50]. Phocine Distemper virus in seals provides an example of disease outbreaks associated with the presence of persistent organic pollutants in the water [55].

Hence, human disturbance can bring about several sources of stress and subsequent immune suppression of wildlife and can thus be a risk factor in outbreaks of both human and wildlife diseases. Researchers need to address every aspect of disease under study through a highly multifactorial approach when it comes to complex disease outbreaks. An example of this is provided by the case of urogenital cancer in sea lions [56]. Researchers in this study examined the host, detecting variations in hormonal receptor expression and variations in the major histocompatibility complex immune system genetics. Considering environmental factors, they also found high concentrations of polycyclic aromatic hydrocarbon (PAHs) and organochlorine pollutants in the sea lion habitats. The authors concluded that the unexpected cancer outbreaks in sea lions were likely a result of the detected multifactorial synergy between host and environmental factors.

Thus, investigating EID systems is complex. Integrative research may entail analyses of hosts and pathogens, along with assessment of potential environmental factors behind disease outbreaks and effects from anthropogenic activities. Investigation of wildlife diseases may eventually point to one or more of these risk factors, acting either singularly or cumulatively towards disease exacerbation and/or propagation. This paper will utilize the framework to cover the case of sea turtle FP, a tumoral disease affecting marine turtles worldwide, and analyze its possible drivers and associated risk factors using our CHANS framework, with a focus on components specific to coastal environments. We have identified all three risk factors from our framework to potentially play a role in FP outbreaks, mainly through the following anthropogenic effects, pollution and climate change, and anthropogenic activities, human encroachment, and urbanization (highlighted in Figure 1). Research can further benefit from our framework by identifying the specific wildlife emerging disease under study and describing which risk factor dynamics may be supporting disease development and outbreaks, as we do here with FP.

## 2. The Case of Sea Turtle Fibropapillomatosis

FP is a neoplastic disease affecting sea turtles worldwide. Along with hunting, habitat degradation, poaching, and pollution, FP has the potential to be a major threat to sea turtle conservation and requires careful research, monitoring, and intervention. FP was first discovered in Florida in 1938 [57]. It has now spread worldwide and has been detected in all seven species of marine turtles. Green turtles (*Chelonia mydas*) are the most affected species, especially juveniles. Recent health assessments from high FP-areas in Florida, for example, have reported consistent FP prevalence up to 50% in sampled juvenile green turtles [58]. FP is much less commonly found in sub-adults and is rare in adult individuals. FP has shown a positive association with herpesvirus infection from Chelonid Herpesvirus 5 (ChHV5) [59]. The virus shows a latent behavior, meaning it infects the host for prolonged periods before disease onset and debilitating issues appear [60,61,62,63]. FP development causes growth of tumoral lesions in affected turtles, concentrated on the skin, mouth, eyes, flippers, and plastron. Severely affected turtles suffer from compromised vision, feeding and mobility, often stranding onshore. Sometimes, lesions affect internal organs such as lungs and kidneys, with fatal outcomes [64].

The cause of FP is multifaceted. In the millions-year-long co-evolution of the sea turtle host and virus, no mutation has been found in ChHV5 to explain the increase in FP prevalence in the last few decades [65]. Therefore, evidence points to environmental factors as potential drivers of outbreaks. No single cause for FP has been discovered, and it is believed that its etiology comprises a complex interaction of multiple environmental factors [66]. This is supported by the fact that juvenile green turtles, the most FP-affected individuals, are nearshore foragers dwelling in coastal areas [67], where heavy anthropogenic alteration has taken place in the last few decades. These environmental changes have occurred in parallel with increasing FP outbreaks in wild sea turtles. Immune system gene variation and higher susceptibility among juvenile individuals might also be risk factors behind FP; however, immunogenetic–environmental interactions are still suggested to potentially be driving disease outbreaks [68]. Previous literature on FP environmental etiology provides evidence that certain impacts from coastal anthropogenic disturbance over time may have been drivers behind exacerbation of this epizootic [66].

### 2.1. Coastal Coupled Human and Natural Systems

Coastal systems under consideration in this review can be classified as CHANS. As of today, only about 15% of the world’s coastlines are considered pristine or minimally affected by anthropogenic pressure [69]. Moreover, 15 out of 20 megacities, defined as cities with a human population density of over 10 million individuals, are located in coastal areas [70]. Urbanization and human activities have transformed a large portion of coastlines into CHANS, in which evolution of coastal habitats intertwines with pervasive anthropogenic influence [71].

**Figure 2:**
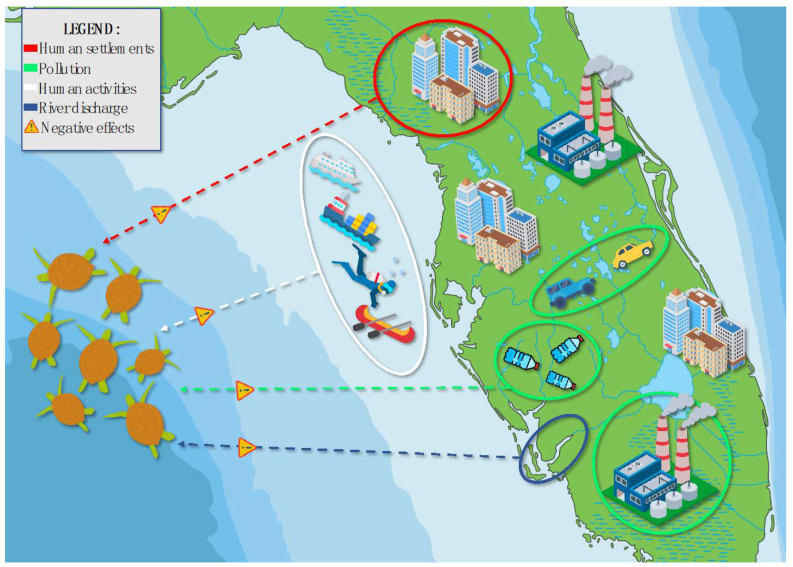
Simplified graphical representation of a coastal Coupled Human and Natural System. The figure aims to represent in simple and comprehensible graphical form the concept of a highly disturbed coastline which creates the potential scenario for Fibropapillomatosis outbreaks. Components include human settlements (red circle), pollution (green circles), human activities (white circle), and river discharge (blue circle). The legend is located on the top left corner. The dashed arrows represent the cumulative negative effects on coastal green turtle populations.

The coastal CHANS analyzed in this paper is simplified for the sake of clarity and is a model representation of many observed systems distributed across urbanized coastal areas (Figure 2). The physical area is generally represented by coastal stretches located in proximity to urban areas, where human settlements gradually encroach onto the shores and waterfronts. Here, just off the coast, is where the city runoff is discharged from nearby river and freshwater systems (blue circle in Figure 2). This discharge includes industrial and domestic sewage, which contains high amounts of contaminants and pollutants (green circles in Figure 2). Human settlements are close to the shore (red circle in Figure 2) and anthropogenic activities at sea are becoming more common both at recreational and industrial scales (white circle in Figure 2). Recreational fishing and snorkeling can be considered of minor impact, if carried out responsibly. However, large-scale fisheries and boating, especially at high speeds, can put turtles in danger and under considerable physiological stress [72]. Juvenile green turtles themselves are members of a CHANS coastal systems, dwelling in the neritic portion of the coast and receiving the cumulative effects of the components mentioned above. Sea turtles are not the only marine animals affected by coastal urbanization, but they will be the species of focus for the purpose of this review. These environmental stressors play a role in immune suppression and wildlife infectious disease outbreaks [73]. Using our CHANS framework, we will discuss how human encroachment (anthropogenic activity), urbanization (coastal settlements), pollution (water quality and pollutant concentration), and climate change (fluctuating seawater temperatures) all have the potential to influence FP in coastal sea turtle populations (human effects highlighted in Figure 1).

### 2.2. Human Encroachment and Urbanization: Anthropogenic Presence and Sea Turtle Health

The level of anthropogenic disturbance experienced in a system can be linked to FP dynamics. Elevated chronic stress conditions resulting from human activities from highly urbanized areas can potentially suppress wildlife immune systems and trigger or exacerbate infection [73]. Sea turtle populations dwelling in urbanized coastal environments have been repeatedly found to present elevated FP prevalence [61,74,75,76,77,78,79]. Habitat loss, human presence, fishing, boating, and encroachment resulting from proximity to anthropogenic areas can significantly stress sea turtle populations. More densely populated areas have been associated with a higher degree of general threat to wildlife conservation, both in terms of infectious disease increase and biodiversity loss due to other types of human activity [80,81,82]. In Puerto Rico, for example, green turtle populations showed over three times higher infection prevalence in more anthropogenically altered areas (21% prevalence) compared to more pristine ones (6% prevalence) [77]. FP incidence has been increasing alongside coastal urbanization, and currently green turtles living near highly urbanized areas are reported to be twice as likely to develop FP [83]. More research from Florida observed a substantially lower FP prevalence in open ocean sites compared to a coastal lagoon (Indian River Lagoon) which is heavily degraded by urban development [84]. Research that identifies and quantifies aspects of specific human activities most harmful to sea turtles would be useful in further FP studies, as human presence likely plays a significant role in the dynamics of this cancerous disease.

### 2.3. Pollution: Role of Water Quality in Sea Turtle Disease

Water quality can also play a major role in aquatic animal health, including sea turtles [85]. River discharge from densely populated areas accounts for high amounts of pollution affecting nearshore water quality, showing a positive correlation with FP prevalence in green turtles [66,86]. The negative impact of environmental contaminants from wastewater on sea turtle health has been discussed since 1995 [59], and there has been increasing evidence and support on the matter [61,74,76,77,78,79,84]. Findings from Brazil, for example, support the association between water quality and FP as degraded habitats reported higher prevalence (58.3%) compared to other areas of the country (15.4%) [74]. Research has looked at contaminants in FP-infected turtles across Hawaii [76,87], Australia [78] and Brazil [88]. Some findings showed blood contamination to correlate with FP viral infection, oxidative stress, and overall poorer health [78,88]. Pollutants such as PAHs, polychlorinated biphenyls (PCBs), and organochlorine pesticides have been previously linked to cancer in aquatic animals, and are classified as some of the most prevalent water pollutants with known oncogenic and tumor initiation effects [89]. Green turtles have been shown to bioaccumulate these classes of oncogenic contaminants, with higher concentrations of PAHs and PCBs found in severely afflicted FP turtles compared to apparently healthy turtles [90,91]. Green turtles are characterized by high site fidelity to their habitats [92,93,94,95] and small juvenile green turtles are expected to spend at least a decade in these nearshore developmental habitats [96], providing a high likelihood for local pollutant concentrations to bioaccumulate and influence resident sea turtle health.

### 2.4. Climate Change: Effect of Fluctuating Temperatures on Coastal Ecosystems

Climate change can also alter wildlife disease dynamics. Extreme temperature changes are not only particularly impactful on ectothermic vertebrates, such as sea turtles, but are also common in nearshore habitats where they live [67,97]. Variation in sea temperature has been indicated to affect FP prevalence [66,98,99,100]. Thermal stress from both high and low temperature extremes influences immune-competence, triggering FP infection, proliferation, and viral shedding [101,102]. Herbst et al. observed that higher water temperatures experimentally promoted FP tumor growth, while lower temperatures delayed their onset [59]. Moreover, in rehabilitation facilities, green turtles have a higher chance of FP development during the warmer months [79]. Therefore, climate change and consequent extreme sea water temperatures are potentially additional anthropogenic contributions behind FP dynamics. Monitoring this sea water parameter and its correlation with FP is important, especially considering the mounting evidence for wide-ranging effects of climate change on the ocean [103].

## 3. The CHANS Novelty of Recognizing Feedback Loops

Feedback loops represent patterns and processes, either positive or negative, that can accelerate or decelerate change in a system in a circular form [5]. These are an inherent part of CHANS and tightly link impacts of human actions on the natural world, and vice versa. Here, we describe a potential feedback loop identified in our FP example.

**Figure 3:**
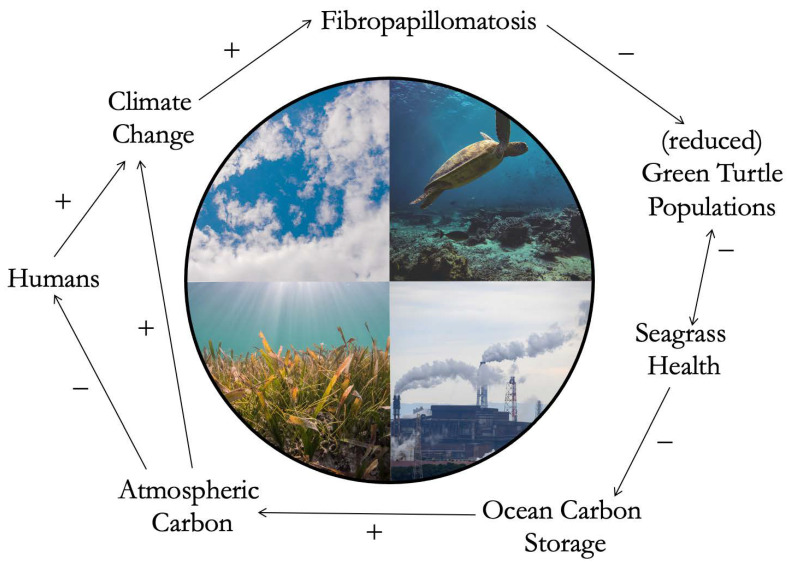
Elaboration of a hypothetical feedback loop characterizing the case of sea turtle Fibropapillomatosis. Each arrow indicates the direction of an effect, positive (+) or negative (–), causing either acceleration or deceleration of a change. Humans (far left) drive climate change, causing fluctuating temperatures which may exacerbate Fibropapillomatosis. Disease occurrence negatively affects green turtle populations. A green turtle population that is reduced due to the negative effects of disease, may in turn affect the health of seagrass meadows. This effect is represented by a double-sided arrow, as loss of seagrass beds can in turn further affect green turtle health. Reduced seagrass health may affect the ability of seagrass to store carbon, potentially increasing the concentration of carbon accumulated in the atmosphere. This may in turn negatively impact human health and also accelerate climate change, creating a loop bisecting the shared human and animal systems. All photographs used in this figure were obtained from unsplash.com (accessed on 30 January 2023, from https://unsplash.com/) and stockvault.net (accessed on 30 January 2023, from https://www.stockvault.net/), under free use, no copyright or permission needed, and Creative Commons licenses.

If we consider climate change and fluctuating ocean temperatures as a key co-trigger candidate behind FP, we can detect a possible feedback loop where human activity simultaneously actively affects and is passively affected by FP (Figure 3). Anthropogenic activity heavily drives climate change [104]. Changing temperatures, as described in the section above, are considered likely co-triggers of FP occurrence among green turtles. Green turtles are herbivorous and feed on a variety of algae and seagrass. In the tropics, where FP mostly occurs [105], green turtles mainly feed on turtle seagrass (*Thalassia testudinum*) when available [106]. In high prevalence, FP may cause decline of the number or frequency of sea turtles feeding on seagrass, compromising their grazing activity, which is essential for maintaining healthy seagrass meadows [107]. As the health of the main dietary item used by green turtle declines, turtle health might further decline, creating a double-effect interaction (double-sided arrow in Figure 3). Furthermore, seagrasses have a hidden and important role—the ability to store large amounts of carbon, up to 35 times higher than tropical forests [108]. With declining seagrass health, and decline of carbon storage capability, carbon concentration in the atmosphere may increase. Atmospheric concentration of carbon dioxide, a primary greenhouse gas produced by the burning of fossil fuels, is concerning when it comes to climate change [109]. Climate change, in turn, negatively affects human health in complex ways, including heatwave-related mortality and an increase in vector-borne infections [110]. This is just one not-so-hypothetical example among the variety of plausible human activities behind FP dynamics that branch into feedback loops. It demonstrates the multifaced nature of the issue, and the high level of interconnectedness between human and natural systems.

## 4. Importance of One Health and Multifocal Interventions

FP is clearly multi-factorial. This disease has not arisen from a recent viral mutation but is a response to an accumulation of external factors acting as co-triggers [64]. With this disease, we are seeing multiple stressor effects on an ecosystem and the organisms living within it. The drastic and rapid changes in coastal ecosystems have triggered a widespread infectious disease in an endangered turtle species. Sea turtles are considered environmental sentinel species. Disruption of ocean health (i.e., temperature ranges, contaminant concentration) may trigger disease outbreaks, thus sea turtle health reflects the health of the environment they live in [111]. In the coastal CHANS, different components can be categorized in different sub-systems (Figure 2). Human settlements, activities and pollution are products of the human sub-system. Green turtle populations belong to the animal sub-system. Rivers and the ocean are the landscape; hence, for simplification, they can be seen as part of the environmental sub-system. If those sub-systems, humans, animals, and the environment were to be perceived as separate, the roots of FP may not be properly targeted. Human settlements can be located on rivers and by the ocean; hence, these components automatically become part of the environmental sub-system as well. Pollution is a human product; however, when released into the environment it disrupts the fauna and flora; hence, it becomes part of both the environmental and animal sub-systems. So on and so forth, every aspect of every sub-system in the coastal CHANS is inherently connected with feedback loops and interactions happening over time. These connections are central to the concept of One Health. The One Health philosophy recognizes the interdependence between the health of human beings, animals, and the environment [112]. In the case of FP, veterinary sciences, molecular biology, virology, and genomics need to cooperate with fields within chemistry and ecology. The philosophy of One Health can be integrated with CHANS principles to better understand FP. When looking at health in a CHANS context, research needs to consider health of humans, animals, and the environment in their coupled system, as well as the interconnection between those realms and how important they are for the functioning of the system itself. As shown in our feedback loop example (Figure 3), human-driven disruption of sea turtle health may alter environmental health, which may eventually lead to disruption of human health, demonstrating that health of humans, animals, and the environment can truly be interdependent. In the case of FP, the health of sea turtles (disease severity and prevalence) and of their environment (temperature, pollution levels) both need to be looked at through a One Health lens. Research needs to seek drivers behind disruption of such health in the tight links that exist between the anthropogenic and the natural world (coastal anthropogenic activity, human-driven stress), as supported by the fundamental principles of CHANS. Further, when reporting data on occurrence, severity, and clinical characteristics of FP, it is important to include data on environmental and water quality to enable detection of possible patterns across disciplines. This is a promising research approach when looking at anthropogenically exacerbated wildlife infectious diseases as complex as the one examined.

Healing sea turtle cancerous lesions is a temporary solution to the broader problem of FP. Treating the symptom will not, in itself, lead to the cure. Solutions for FP can instead be set to target numerous disease risk factors that are each very different in their nature. All considered, human encroachment, urbanization, pollution, and climate change are problems occurring at large scales. With issues as complex and intricate as FP, solutions may only come to light after identifying the main drivers and risk factors (see our framework in Figure 1), and connecting different CHANS sub-systems, along with identifying where interactions among sub-systems and feedback loops occur. Collaboration across disciplines, critical thinking and an open-minded approach are strongly supported when looking at sea turtle FP and similar wildlife diseases. Moving forward, the FP research agenda can focus on the holistic nature of the disease, possible mitigation measures, and the Anthropocene (the Anthropocene currently has no formal status in the Divisions of Geologic Time (https://pubs.usgs.gov/fs/2018/3054/fs20183054.pdf (accessed on 11 April 2023)). It is used here to indicate a time when human activities have significant effects on the global environment.)—rooted drivers that might have caused the emergence and exacerbation of such a poorly understood, yet prevalent epizootic. Topics specific to this area of future research could include but are not limited to (i) quantification of the role of carcinogenic pollutant accumulation in the ocean and associated impacts on turtle FP, as well as on human and whole ecosystem health; (ii) estimation of global mortality rates of FP, including how FP may affect population viability, and consequential cascading effects at the ecosystem level; and (iii) characterization of the routes of viral transmission and spread that can in turn inform the design of potential treatment and preventative measures to mitigate FP infection in wild populations. Failure to understand FP, and other wildlife diseases, as part of complex multifactorial feedback systems, will result in failing to manage and control them and thus in failing to protect human, animal, and environmental health. Multifocal research approaches are a suggested pathway to ensure preservation of sea turtles and effective conservation of our shared ecosystems.

## Data Availability

Data sharing not applicable.
